# Estimating equations for biomarker based exposure estimation under non-steady-state conditions

**DOI:** 10.1186/1476-069X-10-57

**Published:** 2011-06-13

**Authors:** Scott M Bartell, Wesley O Johnson

**Affiliations:** 1 Program in Public Health, University of California, Irvine, CA 92697-3957, USA; 2 Department of Statistics, University of California, Irvine, CA 92697-1250, USA

## Abstract

Unrealistic steady-state assumptions are often used to estimate toxicant exposure rates from biomarkers. A biomarker may instead be modeled as a weighted sum of historical time-varying exposures. Estimating equations are derived for a zero-inflated gamma distribution for daily exposures with a known exposure frequency. Simulation studies suggest that the estimating equations can provide accurate estimates of exposure magnitude at any reasonable sample size, and reasonable estimates of the exposure variance at larger sample sizes.

## Background

Health risks assessment, dietary research, environmental epidemiology, and other endeavors that depend on quantitative exposure estimation are increasingly making use of exposure biomarkers instead of, or in addition to, traditional contact-based exposure estimates [1-4]. Estimation of exposure rates from biological measurements is a particularly challenging problem given the complex relationship between ingested, inhaled, or dermally absorbed chemical exposures and the resulting tissue concentrations over time. Indeed, because many different exposure patterns can lead to the same blood or urine concentration at a given point in time, typical approaches to biomarker based exposure estimation must rely on simplifying assumptions regarding the exposure patterns. In cases where exposures have ceased, such as post-shift or post-retirement studies of occupational exposures, exposure rates are often reasonably assumed to be zero. In other settings, investigators often rely on an assumption that exposure rates are constant over time for each individual.

Unfortunately, virtually all environmental exposures vary in magnitude over time, thereby violating the steady-state model. For example, ingestion occurs intermittently and only during waking hours. These violations can cause substantial errors in biomarker based exposure estimates that rely on a steady-state assumption [5]. The degree of error introduced by the steady-state model is often substantial depending on the elimination rate of the chemical, the frequency of contact, and the variability in exposure over time, even under the highly optimistic assumption that every individual's exact biokinetic parameters are known [5-7]. These results suggest that a substantial portion of observed population variability in mercury biomarker concentrations may result from non-steady-state exposure conditions, rather than being entirely attributable to true differences in individual mercury exposure rates. Exposure measurement error is known to cause bias in epidemiologic dose response modeling, though *post-hoc *methods of adjustment have been proposed [8].

Formal statistical methods for biomarker based exposure estimation that do not rely on steady-state assumptions are needed. Standard Monte Carlo simulation methods have been suggested but are inadequate for inverse estimation problems due to unknown but non-zero correlations [9].

We present a new statistical method for estimating individual exposures to mercury based on individual hair or blood mercury biomarkers and individual exposure frequencies, for a group of people with the same probability distribution of daily exposure magnitudes. Although this method was developed using mercury as a case study, it may be applicable to other toxicants. This method avoids steady-state assumptions and incorporates information from biokinetic models. The new approach utilizes discrete-time approximations to continuous-time biokinetic models and statistical methods based on the theory of estimating equations [10,11].

## Methods

### Simplified Biokinetic Model

The relationship between chemical exposure at the visible exterior boundary of a person and related biomarker measurements is governed by fairly complicated time-dependent processes including absorption across the skin, gastrointestinal tract, or lung epithelium; distribution throughout the body via blood circulation, filtration, metabolism, and/or sequestering by liver and kidneys; and excretion via skin, nails, hair, urine, and feces. The entire system of these processes is referred to as "pharmacokinetics," "toxicokinetics," or "biokinetics."

Mathematically, biokinetic models are typically composed of continuous-time systems of differential equations, with each differential equation representing the rate of change in concentration or mass of a chemical in a particular tissue or organ as the chemical is exchanged with blood, metabolized, or excreted. These biokinetic models are generally not invertible without additional constraints and *ad hoc *methods, due to the dimensional reduction from continuous-time exposure patterns to biomarker measurements at specific time points. In other words, many different exposure patterns can lead to the same biomarker concentration, so it is generally not possible to determine an exact exposure pattern using only biomarker measurements. Instead, biomarker based risk assessments typically rely on the simplifying but unrealistic steady-state assumption, multiplying each individual's biomarker concentration by a steady-state ratio in order to estimate constant exposure rates.

The classic single compartment biokinetic model can be expressed as a differential equation: , where *y*(*t*) is the biomarker concentration at time *t*, *f *is the fraction of ingested mercury present in the blood after absorption across the gastrointestinal tract and equilibration throughout the body, *v *is the volume of blood, *I*(*t*) is the mercury exposure rate at time *t*, and *k *is the excretion rate coefficient for mercury. If *I*(*t*) is constant then *y*(*t*) eventually reaches "steady-state," i.e., and . Thus, the steady-state ratio of *I *to *y *is . An estimate of this steady-state ratio is typically multiplied by each individual's measured biomarker concentration in order to estimate the corresponding exposure rate.

It is more difficult to develop general solutions and frequentist statistical approaches for the single-compartment model under non-steady state conditions. One approach is to use a discrete-time approximation to the continuous-time single compartment biokinetic model [5,12]. This approach allows for formal statistical estimation and incorporates key biokinetic features of the single compartment continuous-time model, while avoiding the unrealistic steady-state assumption. For example, the blood mercury concentration in a person exposed to mercury over a period of time, *t*, may be approximated by:(1)

where *y*_*it *_is the blood mercury concentration in individual *i *on day *t*, *I*_*ij *_is the mercury intake for individual *i *on day *j*, *W*_*ijt *_is the "weight" or influence of the day *j *intake on the day *t *biomarker measurement in individual *i*, and *ε*_*it *_is a statistical error term with expectation 0 and variance *σ*_*ε*_^2^. *W*_*ijt *_expresses the short term absorption and dilution of the mercury into the blood, as well as the elimination of mercury from the body over time. As noted by Sherlock et al. [12] in their least squares estimation of biokinetic parameters from a controlled dosing study, Equation 1 provides a close approximation to a continuous-time one-compartment biokinetic mercury model with first order elimination, provided that , where *f*_*i *_is the fraction of ingested mercury present in the blood of individual *i *after absorption across the gastrointestinal tract and equilibration throughout the body, *v*_*i *_is the volume of blood in individual *i*, and *k*_*i *_is the excretion rate coefficient for mercury. For chronic exposures *t *should be chosen to reflect at least ten half-lives, but needn't include the entire individual history as the earliest exposures will have negligible contributions to the measured biomarker concentration.

A similar approximation can be used for segmental hair analysis:(2)

where  is the hair mercury concentration in individual *i *in the hair segment grown between days *t*_1 _and *t*_2_,  is the influence of the day *j *intake on the hair segment mercury concentration, and  is a statistical error term with expectation 0 and variance *σ*_*ξ*_^2^. The discrete model described by Equation 2 is a close approximation to an appropriate continuous-time biokinetic model with the following expression for the exposure weights:

where *h*_*i *_is the equilibrium ratio for hair to blood in individual *i *[5,13].

### Probability Model for Daily Exposures

Equations 1 and 2 can be used along with modern statistical methods to estimate exposure characteristics from biomarker measurements without imposing steady-state assumptions. For example, the following mixture probability density function allows for intermittent exposures with gamma distributed exposure magnitudes:(3)

where *ω*_*i *_is the exposure frequency (with units of day^-1^), 1_{*S*} _is an indicator function that takes the value 1 when statement *S *is true and 0 otherwise, and *a *and *λ *are parameters describing the gamma distribution. Unlike the lognormal distribution, the gamma distribution can take on a heavily skewed shape or a nearly symmetric shape depending on the values of the two parameters. Here we assume that exposures are independent across days and across individuals. This independence assumption may not be reasonable for individuals who share meals, or for those who obtain multiple meals from the same source item. For example, a person consuming many tuna steaks all cut from the same individual fish should have highly correlated mercury exposures over time.

One important attribute of the zero-inflated gamma distribution shown in Equation 3 is that its expectation and variance are easily computed:  and . For our proposed estimation method, it is useful to reparameterize the zero-inflated gamma distribution using  and . *α *and *β *are the log variance and log mean of the conditional exposure distribution, for days with non-zero exposures. The expectation and variance of the unconditional exposure distribution can then be written as and . We will estimate *α *and *β *rather than *a *and *λ*. This parameterization has two important advantages: 1.) *α *and *β *are unrestricted on the real number line and 2.) *E*(*I*_*ij*_) has only one unknown parameter when individual exposure frequencies can be measured, e.g. by food frequency questionnaires.

In this model all methylmercury exposures within a day are grouped together, so the exposure frequency cannot exceed 1 per day. Although the daily grouping of exposures represented by Equations 1-3 does not capture the full complexity of the exposure profile, the approach is much more realistic than the assumption of constant mercury exposure, is amenable to formal statistical treatment, and can easily be extended to include fixed covariate effects.

We have chosen to group exposures by day, but our model is easily adjusted for grouping into smaller (or larger) time intervals, provided that the biokinetic model weights are selected appropriately. For the best approximation, interval lengths should be small relative to the biological half life of the toxicant being modeled. For example, many solvents are quickly excreted from the body; one day exposure aggregates for these compounds would be too crude to compare with biomarker concentrations, but one to sixty minute intervals might prove reasonable.

## Results

In the case of exposure estimation using biomarker measurements alone, the models formulated by combining the above equations pose a challenge in that the likelihood equations are difficult to obtain due to the convolution of many mixture distributions containing both discrete and continuous components. We first explored normal approximations to the summation in Equation 1, but simulation studies indicate that normality only holds when the exposure frequency is high *and *the variance in daily exposure magnitudes is low, making the normal approximation and classical statistical methods unsuitable for most realistic exposure settings [13]. Instead, we propose estimating equations based on the quasi-likelihood [11]. The estimating equations rely entirely on the expectation and variance of the biomarker measurements in terms of the unknown exposure parameters, bypassing the need for an explicit likelihood equation or even specification of exact probability distributions.

### Algorithm

Estimating equations, particularly in the form of generalized estimating equations [14], have become popular in situations where it is difficult to model complex data, such as correlated data that do not arise from a multivariate normal distribution [11,15]. Although estimating equations do not appear to have been previously applied to non-steady-state biomarker based exposure estimation, the method is quite flexible and appears to be reliable in this setting.

These methods make use of a concept called the "quasi-score function" [10,16]. Consider an *n *× 1 response vector **Y **with expectation vector E**Y **and covariance matrix **V**. Let E**Y **be a function of an unknown *p*-parameter vector **β**, and **D **be the *n *× *p *matrix . The quasi-score function is the *p *× 1 vector.

Under certain conditions "quasi-likelihood" estimation using the quasi-score function shares several key properties with a true likelihood based score function, resulting in similar asymptotic properties to those for maximum likelihood estimates [16]. Quasi-likelihood estimates are obtained by setting each element of the quasi-score function equal to 0 and solving for each element in the vector **β**. These equations are referred to as estimating equations. In practice, the Newton-Raphson method with Fisher scoring is typically used to solve the estimating equations:(4)

starting with , an initial guess for **β**. ,, and E_1_**Y **are the *l*th iterate estimates of **D**, **V**, and E**Y**, respectively, and are all obtained by evaluation at . The algorithm consists of repeated application of Equation 4, incrementing *l *by 1 each time, until  and  only differ by a prespecified negligible amount, at which point  has usually converged to the root  of the estimating equations.  is an estimate of the covariance of , and is easily obtained directly from the algorithm. Although we do not show it here, Equation 4 can usually be written in a more computationally efficient form involving summations of block diagonal elements [15].

In a simple biomarker application, **Y **might represent statistically independent blood mercury measurements *y*_*it *_for *i *= 1, 2, ..., *n *individuals, with one measurement per person. Assume that Equations 1-3 apply and that each individual's biokinetic parameters *k*_*i*_, *f*_*i*_, and *v*_*i *_and exposure frequency *ω*_*i *_are known. Using Equations 1-3, basic mathematical properties of expectations and variances of weighted sums [17], and summation rules for finite geometric series, the vector E**Y **consists of the *n *elements , *i *= 1, 2, ..., *n*, and **V **is a diagonal matrix with diagonal entries . [13]. In this case, *p *= 1 because there is only one unknown parameter *β *in the mean vector E**Y**. Conveniently, here **D **= E**Y**.

In most cases, including our model for biomarkers, **V **depends on additional unknown parameters other than *β*. These additional parameters are denoted by *α*--a scalar in our model, as there is only one unknown variance parameter not contained in the mean vector. There are several different strategies for estimating both *α *and *β*, but the most reliable appears to be the use of alternating estimating equations, whereby a second estimating equation is written for *α*, and the algorithm proceeds with alternating iterative estimation of *α *and *β *[13,15]. The estimating equation for *α *can be written as , where  is the upper diagonal of the estimated covariance matrix  in vector form as , **s **is an "empirical covariance vector" with (*n*^2^+*n*)/2 elements *s*_*ij *_= (*Y*_*i *_- E*Y*_*i*_)(*Y*_*j *_- E*Y*_*j*_) corresponding to the elements of ,  is the (*n*^2^+*n*)/2 length vector of estimates for , and  is the (*n*^2^+*n*)/2 x (*n*^2^+*n*)/2 estimated covariance matrix for the vector **s**.

When the observations are independent (a reasonable assumption for one measurement per person), **V **is a diagonal matrix and many of the elements of  are 0. In this special case, the estimating equations for *α *can be simplified using  with a corresponding *n *length vector for **s**, an *n *length vector for , and an *n *x *n *matrix for . In either case, an iterative equation analogous to Equation 4 can be derived from the estimating equations for *α*. Because there is only one measurement per person, this method relies on the between-subject variability in biomarker measurements for estimation of *α*. This approach is reasonable when subjects have similar exposure sources (e.g., similar types of fish in the diet).

The elements of the vector D* are easily obtained as . An expression for V* is more difficult to obtain without simplifying assumptions. We employ the "independence working matrices" assumption of Prentice and Zhao [15], approximating the elements of V* based on a simplifying assumption of independence and normality among the elements of **Y**. For one measurement per person, this assumption results in a diagonal matrix for V*, with elements (2*V*_11_, 2*V*_22_, ..., 2*V*_nn_). In the case of multiple measurements per person, V* is block diagonal with covariance terms of *V*_*ii*_*V*_*jj *_for measurements at different time points in the same individual. Though these can be crude approximations for V*, resulting estimates of *α *and *β *remain theoretically valid and appear to be reliable at reasonable sample sizes.

It is impossible to estimate both *α *and  from the data alone with only one biomarker measurement per person; in this setting an external estimate of one of the two parameters is needed. In other words, one needs to provide an estimate of either 1.) the variance in exposure magnitude over time, or 2.) the variance of any random errors resulting from sources other than exposure variability (e.g., biomarker measurement error and natural biokinetic variation across individuals. Collecting multiple biomarker measurements per individual may reduce or eliminate the need for specifying external parameter estimates for *α *or .

### Testing and Implementation

In order to examine the performance of the estimating equations under simple conditions, we conducted several simulation studies for the simple setting described above, with only one blood measurement per individual. We generated 10,000 data sets at each of 9 different exposure frequencies and 10 different sample sizes ranging from 2 to 1024. For simplicity we assigned all individuals the same biokinetic parameters throughout the simulations, using the values *f*_*i *_= 0.0475, *v*_*i *_= 5 L, and *k*_*i *_= 0.014 d^-1 ^[1,18] for all *i*, and assumed that *σ*_*ε*_^2 ^was known and relatively small. For ease of interpretation we present results in terms of the mean exposure magnitude, *μ *= *e*^*β*^, and the variance in exposure magnitudes, . All simulations were performed using *μ *= 10 μg d^-1^, *σ*_*g*_^2 ^= 5 μg^2 ^d^-2^, *σ*_*ε*_^2 ^= 0.03^2 ^μg^2 ^d^-2^, and *t *= 1000 d. The algorithm only failed to converge for a few simulated data sets with both low exposure frequency and a sample size less than 10; for the worst case with n = 2 and an exposure frequency of 0.1 the algorithm converged for about 99.4% of the simulated data sets.

**Figure 1:**
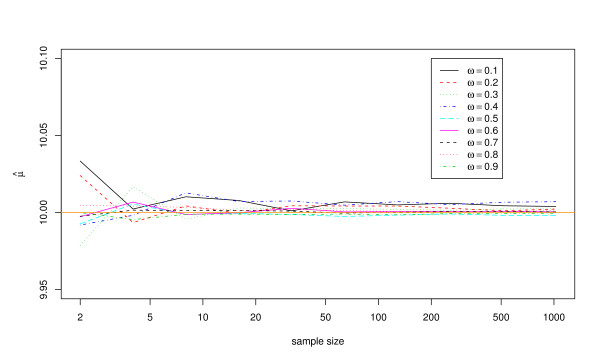
**Mean estimate of the mean exposure magnitude, *μ*, using 10,000 simulated data sets with *μ *= 10, *σ*_*g*_^2 ^= 5, and the estimating equations method**. Results are shown for nine different values of the exposure frequency (*ω*) and ten different sample sizes (*n*).

**Figure 2:**
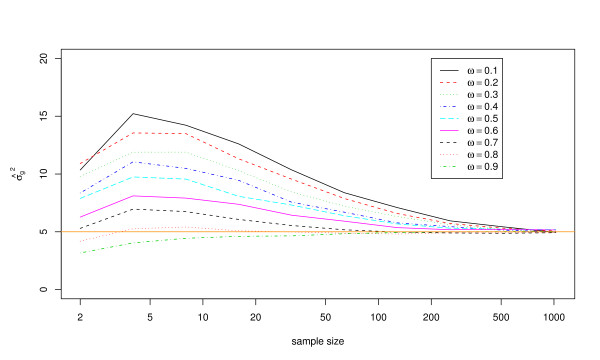
**Mean estimate of the variance in exposure magnitudes, *σ*_*g*_^2^, using 10,000 simulated data sets with *μ *= 10, *σ*_*g*_^2 ^= 5, and the estimating equations method**. Results are shown for nine different values of the exposure frequency (*ω*) and ten different sample sizes (*n*).

Results with regard to potential bias are shown in Figures 1 and 2, and in Tables 1 and 2. Figure 1 suggests that the estimating equations produce unbiased estimates of *μ *regardless of exposure frequency when the model is correctly specified, even if sample sizes are very small. Estimates of *σ*_*g*_^2 ^may be biased at small sample sizes, however, as shown in Figure 2 and Table 2. At small exposure frequencies in particular, *σ*_*g*_^2 ^tends to be overestimated at sample sizes less than about 100 individuals.

**Table 1:** Bias in estimation of the mean exposure magnitude, *μ*

	Exposure Frequency								
Sample Size	0.1	0.2	0.3	0.4	0.5	0.6	0.7	0.8	0.9
2	0.033	0.024	-0.021	-0.008	-0.007	-0.003	-0.002	0.005	0
4	0.002	-0.007	0.017	-0.002	0.006	0.007	0.002	0.004	-0.005
8	0.01	0.004	-0.004	0.013	-0.001	-0.001	0.001	0.003	-0.001
16	0.008	-0.001	0.003	0.007	0	0	0.001	0.001	-0.001
32	0.001	0.004	0.003	0.007	-0.001	0.003	0.001	0.002	-0.001
64	0.007	0.004	0.003	0.005	-0.003	0	0	0.001	-0.001
128	0.005	0.004	0.002	0.007	-0.002	0.001	0	0.001	-0.001
256	0.006	0.003	0.001	0.005	-0.001	0.001	0.001	0.001	-0.001
512	0.004	0.001	0.002	0.007	-0.002	0.001	0.001	0.001	-0.001
1024	0.004	0.002	0.002	0.007	-0.002	0	0	0.001	0

**Table 2:** Bias in estimation of the variance in exposure magnitudes, *σ*_*g*_^2^

	Exposure Frequency								
Sample Size	0.1	0.2	0.3	0.4	0.5	0.6	0.7	0.8	0.9
2	5.34	5.9	4.8	3.36	2.89	1.27	0.3	-0.82	-1.83
4	10.22	8.55	6.89	6.05	4.74	3.11	1.96	0.26	-0.96
8	9.22	8.49	6.89	5.48	4.56	2.91	1.75	0.41	-0.56
16	7.6	6.31	5.29	4.45	3.07	2.38	1.07	0.09	-0.4
32	5.36	4.56	3.44	2.56	2.31	1.43	0.54	-0.02	-0.35
64	3.38	2.88	2.21	1.69	1.39	0.92	0.17	-0.12	-0.17
128	2.09	1.58	1.32	0.77	0.68	0.36	-0.03	-0.19	-0.06
256	0.95	0.72	0.61	0.44	0.31	0.18	-0.1	-0.09	0
512	0.43	0.31	0.2	0.14	0.22	0.21	-0.13	-0.09	0.02
1024	-0.05	0.06	-0.03	0.01	0.19	0.16	-0.08	-0.04	0.04

**Figure 3:**
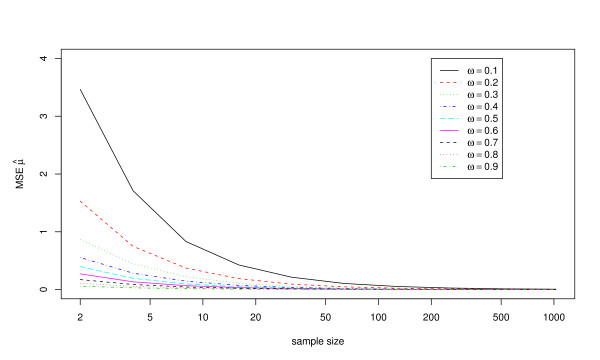
**Mean squared errors for estimates of the mean exposure magnitude, *μ*, using 10,000 simulated data sets with *μ *= 10, *σ*_*g*_^2 ^= 5, and the estimating equations method**. Results are shown for nine different values of the exposure frequency (*ω*) and ten different sample sizes (*n*).

**Figure 4:**
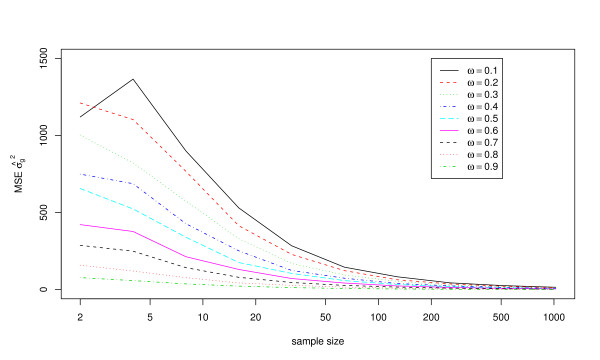
**Mean squared errors for estimates of the variance in exposure magnitudes, *σ*_*g*_^2^, using 10,000 simulated data sets with *μ *= 10, *σ*_*g*_^2 ^= 5, and the estimating equations method**. Results are shown for nine different values of the exposure frequency (*ω*) and ten different sample sizes (*n*).

Figures 3 and 4 show the mean squared errors for the estimates of *μ *and *σ*_*g*_^2^, respectively. These results show good accuracy of the estimates of *μ *at sample sizes of at least 20 for all exposure frequencies, and good accuracy at even smaller sample sizes for larger exposure frequencies. In contrast, a large sample size may be required to ensure that *σ*_*g*_^2 ^is estimated accurately by a single study.

95% confidence intervals were also constructed for *μ *and *σ*_*g*_^2 ^using the estimated covariances from the simulations, assuming approximate normality of the estimators and using the plug-in method (exponentiation of the 95% confidence bounds for *β *and *α*). Table 3 shows the actual coverage rates for nominal 95% confidence intervals for *μ *at each simulated exposure frequency and sample size. Coverage rates are generally close to the nominal 95% value for *μ*, though they were as low as 91% in some cases for sample sizes less than 5. In contrast, coverage rates for *σ*_*g*_^2 ^exceeded 99% for all simulated conditions, indicating that the confidence intervals for *σ*_*g*_^2 ^are overly conservative. The reasons for this are unclear, but may be due to the crude approximation of V* used in our algorithm and/or apparent departures from normality. Further methodological work may be useful if *σ*_*g*_^2 ^is an important target for hypothesis testing and inference, in addition to estimation.

**Table 3:** Coverage of nominal 95% confidence intervals for the mean exposure magnitude, *μ*

	Exposure Frequency								
Sample Size	0.1	0.2	0.3	0.4	0.5	0.6	0.7	0.8	0.9
2	94.5%	94.7%	94.8%	94.8%	94.3%	94.0%	94.1%	93.7%	91.0%
4	94.9%	95.0%	94.8%	95.0%	94.8%	94.6%	94.7%	93.8%	91.9%
8	95.3%	95.5%	94.9%	95.2%	94.9%	94.4%	94.6%	94.4%	93.2%
16	95.3%	95.3%	95.2%	94.9%	95.2%	94.9%	94.8%	94.0%	93.4%
32	95.1%	95.5%	95.3%	95.1%	95.1%	94.7%	94.8%	94.4%	94.2%
64	95.1%	95.2%	95.7%	94.8%	94.9%	94.5%	94.5%	94.5%	94.3%
128	95.2%	94.9%	95.1%	95.1%	95.2%	94.6%	94.8%	94.4%	94.9%
256	95.2%	95.1%	95.2%	94.9%	95.1%	95.2%	94.9%	95.0%	94.9%
512	95.5%	95.3%	95.2%	94.5%	94.9%	94.8%	94.8%	95.0%	95.4%
1024	95.1%	94.9%	95.0%	94.3%	95.2%	95.2%	94.9%	94.9%	94.7%

## Discussion

The simulation studies indicate that the estimating equations are quite reliable for estimation of mean exposure magnitudes even at fairly low sample sizes, but that the variance in exposure magnitudes may be difficult to estimate at low sample sizes when the exposure frequency is low. Confidence intervals are also readily obtained and reasonable accurate for mean exposure magnitudes, but may be overly conservative for the variance in exposure magnitudes.

It is worth noting that the steady-state method assumes a constant exposure rate, implying that the exposure frequency is 100% and that the exposure variance is 0. Observed between-individual variability in biomarker measurements is therefore assumed to reflect true individual differences in exposure rates, rather than day-to-day variability in exposures. The steady-state method has previously been shown to be imprecise in estimating individual exposure rates when day-to-day variability exists [5]. If the correct value is used for the steady-state ratio *b*, the steady-state method estimates *μ *with a bias of  and a precision of  when applied to an individual with non-steady-state zero-inflated exposures [13]. In our simulation setting, the bias of the steady-state estimate for *μ *is therefore approximately 10(*ω*_*i *_- 1) and the variance of the steady-state estimate (for each individual) is approximately . Thus, the standard errors for the steady-state estimate range from 0.26 to 0.44 in our simulation setting. Although the estimating equations appear to produce unbiased estimates for *μ *in nearly all of our simulations, clearly outperforming the steady-state estimate at all nine exposure frequencies in this setting, it is worth noting that a simple modification of the steady-state estimator from to  produces nearly unbiased estimates for large *t*, and averaging those estimates for groups of individuals with similar exposure sources would improve the precision of the estimate of *μ*. With these modifications, the steady-state method might be a reasonable approach for estimating group-averaged exposure rates when the exposure duration is long, provided that exposure variability is only a nuisance instead of a target for estimation.

Unlike steady-state methods, the estimating equations provide estimates and standard errors for both exposure magnitude parameters when *σ*_*ε*_^2 ^is negligible or can be estimated from external data. Statistical theory and our simulations suggest that the estimating equations estimates of *β *have approximately normal distributions at sample sizes above 20 or 30. At high exposure frequencies, even fewer biomarker samples may be necessary in order for the estimates to be normal. Unfortunately, estimates of *α *generated in the simulation studies exhibited fairly strong departures from normality even with hundreds of samples, suggesting that the usual asymptotic normal confidence intervals for *α *might not be appropriate for typical biomarker studies. If confidence intervals for *α *are desired, jackknife or bootstrap procedures might provide more accurate results. However, such estimates still depend on the correct specification of *σ*_*ε*_^2^. If variability in daily exposure magnitudes is an important target for inference, we recommend that multiple biomarker measurements be obtained for each individual. Carefully structured repeated biomarker measurements and duplicate samples may provide a means to simultaneously estimate both *α *and *σ*_*ε*_^2^.

It is possible to extend the estimating equations to handle multiple biomarkers per individual. For example, to extend the simple model to the case with two biomarkers per individual collected at times *t*_1 _and *t*_2_, the biomarker vector **Y **is doubled in length, E**Y **is identical for each pair of measurements from the same individual, and **V **becomes larger due to the additional elements describing the covariance between repeated measurements in the same individual [13]:

Further study is needed to determine whether this approach or alternatives such as population-averaged generalized estimating equations are more reliable in this setting.

Incorporation of interindividual variability in the biokinetic parameters is another goal for extension of these methods. Although additional variance parameters would be difficult to estimate with only one measurement per person, biokinetic parameters that vary across individuals but are stable over time might be estimable from repeated biomarker measurements. In principle these extensions might be accomplished using estimating equations, but Bayesian approaches become more attractive with increasing model complexity [13].

## Conclusions

Direct exposure measurements such as those obtained by duplicate diet studies are often prohibitively expensive for chronic exposure situations such as mercury exposure via ordinary seafood consumption. Indirect estimation using exposure biomarkers is an informative and less expensive approach, but such an exercise should be recognized as a statistical problem whereby the unknown exposure parameters are estimated based on a theoretical model relating the unknown exposures to the observed biomarker measurements.

Our proposed estimating equation approach to biomarker based exposure assessment represents a compromise between the steady-state model, which is overly simplistic but still widely used because of its practicality, and fully detailed biokinetic models that are somewhat impractical for use in formal statistical estimation with ongoing exposures. Our methods have some clear advantages for mercury exposure estimation compared to the steady-state model, due to the more realistic model and the ability to do hypothesis testing and statistical inference. We also believe it may be a valid approach for other chemicals exhibiting first-order biokinetics, provided that the discrete-time unit length is selected to be short relative to the biological half-life. However, its current implementation is based on somewhat restrictive assumptions, including that biokinetic parameters are known and constant across individuals, that measurement error is negligible or can be estimated externally, that individuals can be grouped according to similar exposure distributions, that exposures are independent across days and individuals, and that individual exposure frequencies can be accurately measured.

Future work should assess the performance of both steady-state and non-steady-state methods when these assumptions are violated, as well as extending these methods towards less restrictive assumptions.

## List of Abbreviations

EPA: Environmental Protection Agency

## Competing interests

The authors declare that they have no competing interests.

## Authors' contributions

SB conceived of the study, developed the models, derived the estimators, performed the simulation studies, and drafted the manuscript. WJ contributed to the methods development and helped to draft the manuscript. Both authors read and approved the final manuscript.
